# Organelle interplay—peroxisome interactions in health and disease

**DOI:** 10.1002/jimd.12083

**Published:** 2019-04-16

**Authors:** Michael Schrader, Maki Kamoshita, Markus Islinger

**Affiliations:** ^1^ College of Life and Environmental Sciences, Biosciences University of Exeter Exeter UK; ^2^ Institute of Neuroanatomy, Center for Biomedicine and Medical Technology Mannheim, Medical Faculty Manheim University of Heidelberg Mannheim Germany

**Keywords:** ACBD5, endoplasmic reticulum, fatty acid beta‐oxidation, lipid droplets, lipid metabolism, lysosomes, membrane contact sites, mitochondria, organelle interaction, peroxisomes

## Abstract

Peroxisomes are multifunctional, dynamic, membrane‐bound organelles with important functions in cellular lipid metabolism, rendering them essential for human health and development. Important roles for peroxisomes in signaling and the fine‐tuning of cellular processes are emerging, which integrate them in a complex network of interacting cellular compartments. Like many other organelles, peroxisomes communicate through membrane contact sites. For example, peroxisomal growth, positioning, and lipid metabolism involves contacts with the endoplasmic reticulum (ER). Here, we discuss the most recent findings on peroxisome‐organelle interactions including peroxisome‐ER interplay at membrane contacts sites, and functional interplay with mitochondria, lysosomes, and lipid droplets in mammalian cells. We address tether proteins, metabolic cooperation, and the impact of peroxisome interactions on human health and disease.

## 1 INTRODUCTION

The presence of membrane‐bound organelles is a hallmark of eukaryotic cells. Those distinct compartments create optimized micro‐environments to promote a myriad of metabolic reactions required to sustain life. For the entire cell to function as a unit, a coordinated interplay between specialized organelles must occur. Studies in the last decade have revealed that this is often mediated through inter‐organellar membrane contacts, whereby two (or more) organelles come into close apposition^1^, ^2^ allowing the exchange of metabolites, lipids, and proteins. Studies in mammalian and yeast cells have shown that membrane contacts are much more frequent than previously assumed and not limited to the endoplasmic reticulum (ER),^3^, ^4^ which changes the historical view of organelle contacts and communication. Inter‐organellar connections depend on interacting proteins which act as tethers to bridge the respective organelle membranes. Although closely opposed and tethered, membranes at contact sites are not fused, and usually spaced at 10 to 30 nm, so that ribosomes are excluded from the ER surface at ER contact sites.^1^, ^5^ Recent studies in the rapidly growing field of membrane contacts and organelle interaction have identified molecules associated with several membrane contact sites (MCSs) and revealed their functions, including lipid and ion transport between organelles, as well as organelle positioning and division (reviewed in References 6, 7, 8, 9, 10, 11, 12). It is becoming evident that MCSs are central to normal cell physiology. Moreover, several MCS proteins have been linked to various diseases.^12^, ^13^, ^14^, ^15^

**Figure 1:**
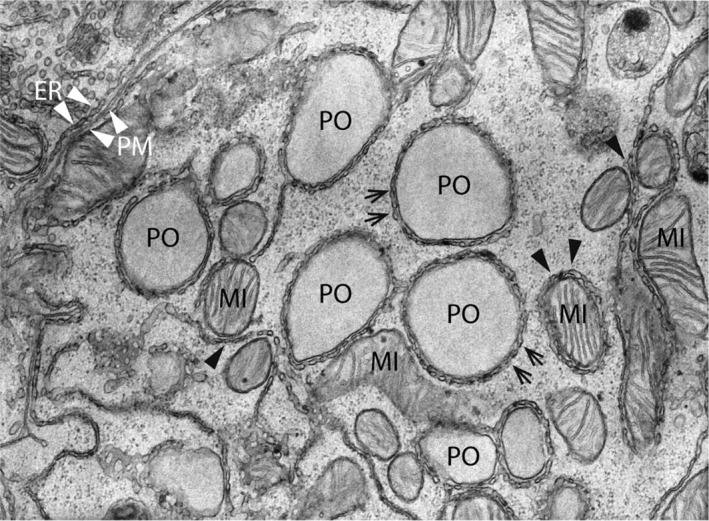
Electron micrograph of organelle contact sites in the rat liver. The center of the image shows five peroxisomes (PO), which are surrounded by a reticular network of smooth ER tubules (arrows). Furthermore, several mitochondria (MI) with ER MAMs can be found (black arrowheads). Note an elongated mitochondrion (center) in direct apposition to the PO‐ER contacts suggesting the existence of functionally relevant organelle triple contacts. ER—plasma membrane (PM) contacts are also observed (upper left corner; white arrowheads). Magnification: ×25 000 (kindly provided by W. Kriz, University of Heidelberg, GER)

**Figure 2:**
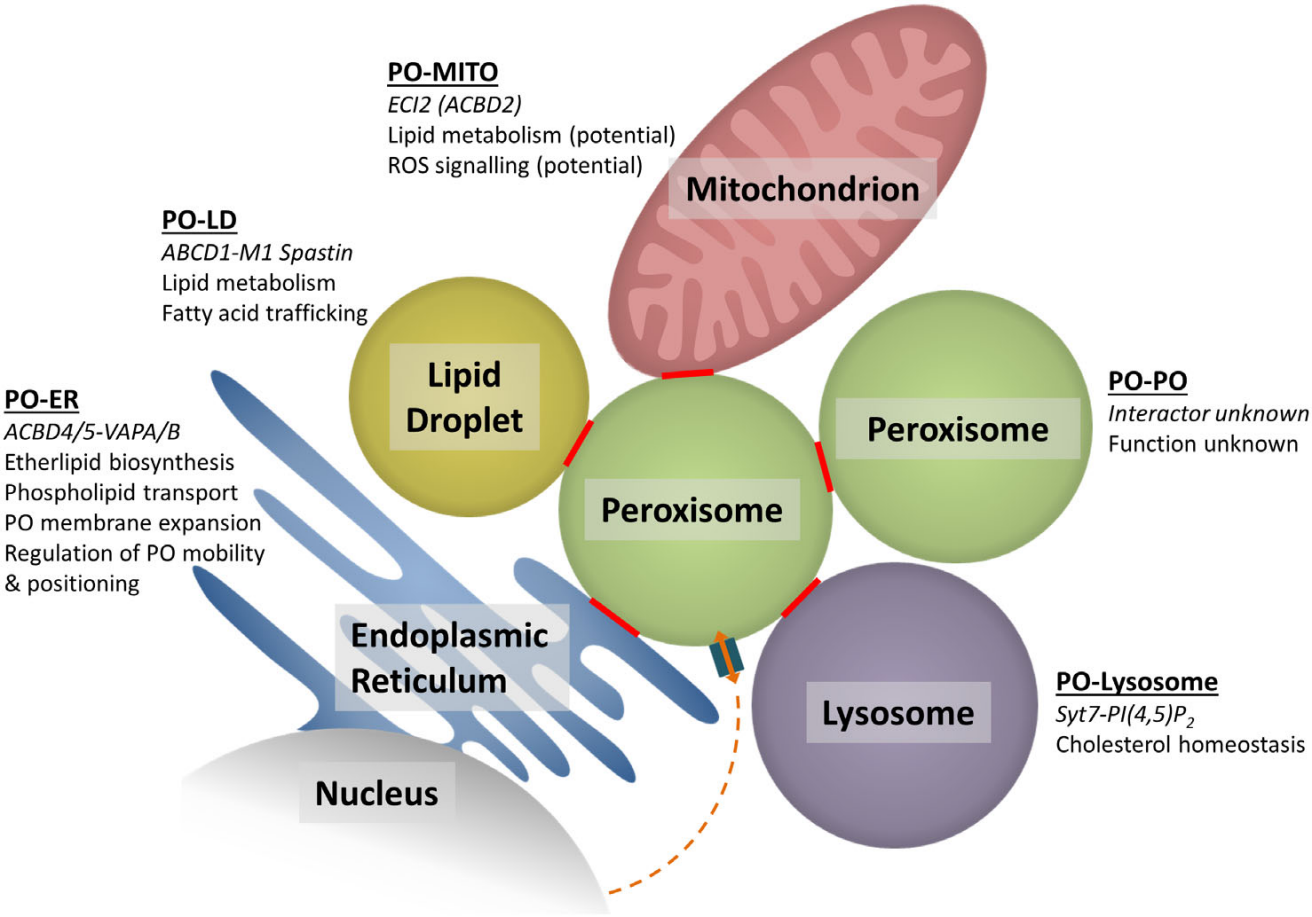
Peroxisome‐organelle MCSs in mammalian cells and their suggested functions. Communication between PO and the nucleus is also indicated. ER, endoplasmic reticulum; LD, lipid droplet; MITO, mitochondrion; PO, peroxisome

Peroxisomes are dynamic, multifunctional organelles with an oxidative type of metabolism. They represent key metabolic organelles with important cooperative roles in the metabolism of cellular lipids and reactive oxygen species (ROS), which renders them essential for human health and development.^16^ Due to their central metabolic role, peroxisomes have to interact and to cooperate with many organelles involved in cellular lipid metabolism such as the ER, mitochondria, lipid droplets, lysosomes, and phagosomes^9^, ^17^, ^18^ (Figures 1 and 2). There is also functional interplay between peroxisomes and the nucleus^19^, ^20^ which may also involve signaling via H_2_O_2._ 21 Mammalian peroxisomes contribute to the breakdown and detoxification of fatty acids (via fatty acid α‐ and β‐oxidation), the synthesis of ether‐phospholipids (eg, myelin sheath lipids), bile acids and docosahexaenoic acid, glyoxylate metabolism, amino acid catabolism, polyamine oxidation, and ROS/reactive nitrogen species (RNS) metabolism. Effective degradation of fatty acids by peroxisomal β‐oxidation, for example, requires a highly interconnected metabolic network involving mitochondria and the ER as discussed in detail in Section 2.4.^18^ Effective bile acid synthesis requires metabolic cooperation between the cytosol, mitochondria, ER, and peroxisomes,^18^ whereas effective ether‐phospholipid synthesis or the synthesis of docosahexaenoic acid depends on the metabolic interplay of peroxisomes and the ER^18^ (see Section 2.5). In addition to their metabolic functions, peroxisomes represent important intracellular signaling platforms modulating physiological and pathological processes such as innate immunity, inflammation, and cell fate decision.^17^, ^22^, ^23^

For decades, it has been observed in electron microscopy studies that peroxisomes are often found juxtaposed to other organelles, in particular the ER, mitochondria, and lipid droplets (Figure 1). These observations provided the first indication that peroxisomes may form MCSs with neighboring organelles. In recent years, several peroxisome‐organelle contact sites have been reported in yeast and mammals (reviewed in References 9, 11, 24, 25, 26 (Figure 2). In this review, we will discuss recent exciting findings on molecules involved in peroxisome‐organelle contact sites, their functions, impact on cell physiology, and—as far as revealed—their link to disease. We particularly focus on peroxisome‐organelle interactions in mammalian/human cells, but where appropriate also refer to recent discoveries in yeast.

## 2 PEROXISOME—ER CONTACTS AND FUNCTIONAL INTERPLAY

Among the peroxisome‐organelle interactions, the most remarkable one is the intricate interplay between peroxisomes and the ER in mammalian cells. In ultrastructural studies, peroxisomes have often been observed in close proximity to the ER, frequently even tightly wrapped in ER cisternae^27^ (Figure 1). Short electron dense cross‐bridges between isolated peroxisomes and associated ER‐segments were also reported.^28^ These connections remain intact even after subcellular fractionation and gradient centrifugation suggesting an intimate, physical interaction. Despite the decades that have passed since peroxisome‐ER associations were first observed, the identification of the molecular machinery involved and its physiological function has only recently begun (Figure 2).

**Figure 3:**
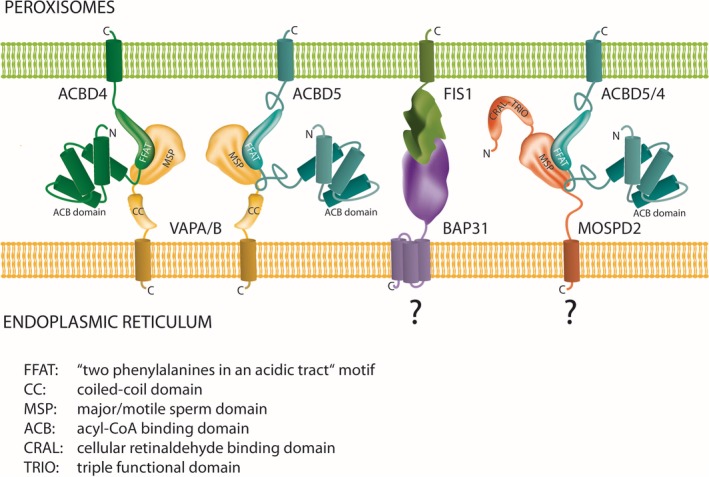
Tethering complexes at peroxisome‐ER MCSs. All hitherto identified and potential tethering complexes connect organelle membranes via protein‐protein interactions. The C‐tail‐anchored proxisomal membrane proteins ACBD4 and ACBD5 possess FFAT‐like motifs in their middle domain which interact with N‐terminal major sperm‐binding (MSP) domains of ER‐resident VAPA and VAPB. With MOSPD2, another ER‐resident protein with an MSP domain was recently identified, which interacts with a variety of tether proteins containing FFAT motifs.^36^ MOSPD1 is another MSP‐domain containing protein with a proposed ER localization.^37^ Interaction of MOSPD proteins with ACBD4/5 has not yet been experimentally verified. The tail‐anchored membrane protein FIS1 was identified in a tethering complex with ER‐resident BAP31.^38^ As FIS1 also localizes to peroxisomes,^39^ the FIS1‐BAP31 tether may also contribute to peroxisome‐ER MCSs

A machine learning prediction approach combined with mutational analyses led to the discovery of new tail‐anchored adaptor proteins at peroxisomal membranes (and other subcellular organelles).^29^, ^30^ These findings then revealed the molecular machinery of the first peroxisome‐ER MCSs in mammalian cells: Acyl‐coenzyme A (CoA)‐binding domain protein 5 (ACBD5), a tail‐anchored peroxisomal membrane protein,^31^, ^32^, ^33^ which mediates peroxisome‐ER MCSs by binding through its FFAT‐like motif (two phenylalanines [**FF**] in an **a**cidic **t**ract) to VAP proteins (vesicle‐associated membrane protein [VAMP]—associated proteins) (VAPA, VAPB) at the ER^34^, ^35^ (Figures 2,3). ACBD proteins comprise a large multigene family of intracellular lipid‐binding proteins, which are found in all eukaryotes and are ubiquitously expressed in mammalian tissues. ACBDs are involved in cellular signaling and lipid metabolic pathways, thus controlling energy regulation.^40^ VAPs are highly conserved tail‐anchored ER membrane proteins (VAPA and VAPB in animals, Scs2 and Scs22 in yeast). They function as ER‐adaptor proteins involved in inter‐organellar lipid exchange, MCS formation, and membrane trafficking.^41^ VAPs contain a major sperm protein (MSP) domain that interacts with the FFAT motif of protein partners such as ACBD5 located on the opposing membrane^42^ (Figure 3). VAPs are involved in ER‐MCS formation with multiple organelles; for example, VAP‐PTPIP51 interactions bridge contacts between ER and mitochondria.^43^

Quantitative electron microscopy revealed that co‐expression of ACBD5 and VAPB increased the number and surface of peroxisome‐ER MCSs, supporting a function of this interaction in peroxisome‐ER tethering.^34^ An ACBD5 mutant with an altered FFAT motif was, however, unable to increase peroxisome‐ER MCSs when co‐expressed with VAPB. In line with this, depletion of either ACBD5 or VAPA/VAPB reduced peroxisome‐ER MCSs.^34^, ^35^ Overall, ACBD5‐VAPB‐mediated peroxisome‐ER contacts fulfill the following properties, which have been defined to aid in the identification of bona fide MCSs^1^, ^5^: (a) the tethered organelle membranes must be in close apposition, typically within 30 nm, (b) the membranes do not fuse, (c) specific proteins (and/or lipids) must be enriched at the MCS. With respect to (d), “MCS formation must affect the function or composition of at least one of the tethered organelles,” several functional alterations have been revealed, which will be addressed in the following (see Sections 2.1‐2.5).

### 2.1 Peroxisome‐ER MCSs and their role in peroxisomal membrane expansion and phospholipid transfer

#### 2.1.1 The lipid composition of peroxisomes

While there is a variety of publications aimed at defining the peroxisomal matrix and membrane proteome,^44^ there is an evident scarcity of studies examining the lipid composition of peroxisomal membranes. Two older studies determined the phospholipid composition of peroxisome membranes from *Saccharomyces cerevisiae* and rat liver.^45^, ^46^ Both membranes exhibit similar phospholipid compositions of approximately 50% phosphatidylcholine (PC), 25% phosphatidylethanolamine (PE), 5% phosphatidylserine (PS), with an exception for phosphatidylinositides (PI), which appear to be significantly higher in the yeast (16% compared to 5%). In this regard, peroxisome membranes have a typical phospholipid composition of intracellular membranes (see Table 1), while plasma membranes are richer in PS (25%) but less abundant in PE (16%) and PC (36%) (values for human embryonic kidney 293 cells (HEK)).^47^ Plasma membranes are also highly enriched in cholesterol comprising up to 65% to 80% of the total cellular content, while minute amounts of 0.5% to 1% are found in the ER membrane^48^ and an intermediate proportion of 5% has been estimated for peroxisomes.^49^ PIs represent a heterogeneous group of important cellular signaling molecules which are distinguished by the phosphorylation state of their inositol head group and show a compartment‐specific distribution. The phosphorylation state determines their function, modulates their interaction partners and thus the signaling pathways they participate in. Peroxisomes have been found mainly enriched in phosphatidylinositol 4‐phosphate (PI(4)P), PI(4,5)P_2_, and PI(3,5)P_2_ bearing only low amounts of PI(3)P.^50^ With regard to the differences in lipid membrane composition between individual organelles and the plasma membrane it is tempting to speculate on corresponding differences in membrane physiology. Generally the overt low cholesterol and sphingolipid concentrations in most intracellular membranes render those less densely packed and rigid than the plasma membrane, which may reflect their reduced need to resist mechanical stress.^51^ PE is considered to be required for membrane bending and curvature.^52^ Thus, the relatively high concentrations in the membranes of peroxisomes and mitochondria may mirror a highly dynamic nature of the membranes required for rapid fission or in case of mitochondria as well fusion events. However, as intramolecular composition in fatty acids (grade of saturation, chain length), asymmetrical lipid distribution between outer and inner leaflets and influence of membrane proteins are further important determinants of biomembrane properties,^51^ the current lack of detailed information on the peroxisomal membrane composition precludes any reliable predictions on architecture and function.

**Table 1:** Relative concentrations of membrane lipids in different organelles from yeast and rat liver

	Rat liver^a^			Yeast^b^		
	Mito (%)	ER (%)	Peroxisome (%)	Mito (%)	ER (%)	Peroxisome (%)
PE	29	19	27.5	26.5	33	23
PC	42	55.5	56.5	40	51	48
PS	7.2	7.2	3.0	3.0	6.6	4.5
PI	3.7	10.5	4.7	14.6	7.5	16
CL	7.6	0	0	13.3	6.6	7
SPM	1.6	4.2	3.7	n.d.	n.d.	n.d

Abbreviations: CL, cardiolipin; n.d., not determined; PC, phosphatidylcholine; PE, phosphatidylethanolamine; PI, phosphatidylinositol; PS, phosphatidylserine; SPM, sphingomyelin.

a Values taken from Reference 45.

b Values taken from Reference 46.

#### 2.1.2 Peroxisomal growth and division

Peroxisomes are dynamic organelles which can multiply by growth and division of pre‐existing organelles in a multistep process involving membrane elongation, constriction and final membrane scission (reviewed in References 20, 53, 54). The peroxisomal membrane protein PEX11β is a key factor in the regulation of peroxisome number in mammals, as it is associated with all steps of peroxisomal growth and division.^55^, ^56^, ^57^, ^58^ PEX11β mediates membrane growth by remodeling, deforming, and elongating the peroxisomal membrane prior to fission. This involves PEX11β oligomerization and interaction with membrane lipids through its N‐terminal amphipathic helixes.^59^, ^60^, ^61^ Besides acting as a membrane‐shaping protein, PEX11β is also involved in the assembly of the division machinery by interacting with the C‐tail‐anchored membrane adaptors FIS1 (fission protein 1) and MFF (mitochondrial fission factor), which recruit the dynamin‐related fission GTPase DRP1 (DNM1L) to the peroxisomal membrane (reviewed in Reference 20). Finally, PEX11β functions as a GTPase‐activating protein (GAP) for DRP1 during peroxisomal fission.^58^ An important discovery in the field was that peroxisomes share several proteins of their division machinery (eg, FIS1, MFF, DRP1) with mitochondria (see section 3). Recently, dynamin‐based ring motive‐force organizer 1 (DYNAMO1), a 17‐kDa nucleoside diphosphate kinase‐like protein, was identified as a component of the division machinery of peroxisomes and mitochondria using the unicellular red alga *Cyanidioschyzon merolae*.^62^ DYNAMO1 converts ATP to GTP, and is suggested to fuel membrane fission by local GTP generation. DRP1 or MFF deficiency in patients results in highly elongated peroxisomes (and mitochondria), which are unable to divide.^63^, ^64^, ^65^

Elongation and growth of the peroxisomal membrane requires lipids, which have to be provided by the ER as peroxisomes lack an appropriate enzyme inventory for autonomous synthesis.^46^, ^66^ Indeed, a direct, nonvesicular phospholipid transfer between the ER and peroxisomes has been verified using an in vitro system based on *S. cerevisiae* mutants.^67^ The authors showed that purified peroxisomes receive PS from purified microsomes without requiring ATP or the addition of cytosol. Unlike mitochondria, peroxisomes do not fuse.^68^, ^69^ Therefore, the massive elongation of peroxisomes after loss of DRP1 or MFF implies a constant transfer of phospholipids from the ER to peroxisomes. In line with this, depletion of ACBD5 or VAPs to disrupt peroxisome‐ER MCSs in patient fibroblasts deficient in MFF reduced membrane expansion, resulting in the formation of shorter peroxisomal membrane tubules and spherical organelles.^34^ Similar results were obtained in DRP1‐depleted HeLa cells after silencing of ACBD5 or VAPs.^35^ The elongated mitochondrial morphology was unaltered, demonstrating that this effect was specific for peroxisomes. Interestingly, expression of an artificial peroxisome‐ER tether restored membrane expansion after ACBD5 depletion in MFF‐deficient fibroblasts. This implies that ACBD5 is likely not actively involved in the transfer of membrane lipids between both organelles.^34^ However, these findings strongly support a role of ER‐peroxisome MCSs in phospholipid transfer for peroxisome membrane expansion and biogenesis.

#### 2.1.3 Lipid transport systems

How lipids are transferred between the ER and peroxisomes is an exciting and challenging open question which needs to be addressed in future studies. As outlined above, a dominant role for ER‐derived pre‐peroxisomal vesicles in lipid transport to peroxisomes may be unlikely, in particular as approximately 70% to 80% of peroxisomes in mammalian cells associate with the ER allowing direct lipid transfer. For other MCSs, several families of lipid transfer proteins have been identified, which are known to facilitate glycerophospholipid, sphingolipid, or sterol transport between membranes—the oxysterol‐binding proteins (OSBP) and OSBP‐related proteins (OSRP), the synaptotagmin‐like mitochondrial‐lipid‐binding domain proteins (SMP), the phosphoinositol transfer proteins (PTIP), and the START domain‐related proteins (STARD).^70^, ^71^ Mechanistically, these proteins can facilitate lipid transfer via two different modes; either anchored proteins bridge donor and acceptor membranes and possess a flexible lipid binding domain for the transfer, or soluble proteins act as shuttle between both membranes. Proteins of the former type can be directly inserted into an organelle membrane by hydrophobic transmembrane helices or specifically interact with integral membrane proteins or lipid species at the organelle surface. One example is the OSBP protein, which is anchored to the ER membrane via interaction with VAPA and to the trans‐Golgi network (TGN) through interaction with ADP‐ribosylation factor 1 (ARF1) and PI(4)P^72^; another example are the extended‐synaptotagmins (E‐Syt 1‐3), which possess a membrane‐spanning domain to anchor at the ER and a C‐terminal C2 domain capable of binding PIPs at the plasma membrane.^73^ Proteins of the latter type are the phosphatidylinositol transfer proteins PITPα and β, which possess a lipid binding pocket to shield the hydrophobic cargo PI or PC while possibly shuttling from the donor to the acceptor membranes through the cytosol.^74^ No peroxisome‐specific lipid transfer proteins of either type have been identified yet. However, it is likely that distinct members of these protein families reside on peroxisomes awaiting identification.

### 2.2 Peroxisome‐ER MCSs and their role in peroxisome mobility and distribution

Interestingly, the ACBD5‐VAP tether also controls peroxisome movement and positioning. Automated detection and live tracking of peroxisomes in human skin fibroblasts after depletion of ACBD5 using a customized in‐house algorithm revealed an increase in the number of moving peroxisomes as well as in peroxisome displacements.^34^, ^75^ A significant increase in both peroxisome mobility and diffusion coefficient was also observed in COS7 cells after depletion of both VAPs or ACBD5 alone.^35^ These findings indicate that MCSs can modulate organelle mobility, positioning, and distribution and thus the spatio‐temporal organization of the cell. Although we do not know much about how peroxisomal function depends on the spatial distribution of peroxisomes, a link between peroxisome positioning and cell fate decisions in skin epithelia has recently been revealed.^22^ It is therefore possible that MCSs can affect cellular homeostasis by controlling organelle positioning.

In cultured mammalian cells, peroxisomes are often uniformly distributed, and only about 10% of the peroxisome population is moving in a microtubule‐dependent manner at any one time.^76^, ^77^ Ultrastructural analyses of cultured mammalian cells to quantify peroxisome‐ER associations using unbiased spatial stereology revealed that 70% to 80% of the peroxisomes tightly associate with the ER under standard conditions.^34^ A prominent peroxisome‐ER interaction likely restricts peroxisome mobility and would explain why only a small population of peroxisomes has been observed to move in mammalian cells.

Recently, a role for the mitochondrial Rho GTPase MIRO1 in the recruitment of microtubule‐dependent motor proteins to peroxisomes was revealed.^25^, ^26^, ^78^ MIRO1, a tail‐anchored membrane protein initially described as a mitochondrial membrane adaptor for kinesin, also targets peroxisomes and contributes to peroxisome distribution and microtubule‐dependent motility.^29^, ^30^, ^34^ We recently generated a MIRO1 protein which is exclusively targeted to peroxisomes (MIRO1‐Pex). As MIRO1‐Pex recruits the motor protein kinesin to peroxisomes, it can be used as a tool to exert (kinesin‐driven, microtubule plus end directed) pulling forces at peroxisomes.^25^, ^26^ When expressed in COS7 cells, peroxisomes redistribute to the cell periphery (where microtubule plus ends are located). Surprisingly, MIRO1‐Pex expression in human skin fibroblasts did not result in a redistribution of peroxisomes, but MIRO1‐Pex‐mediated recruitment of kinesin and subsequent motor pulling forces rather promoted the division and proliferation/multiplication of peroxisomes.^25^, ^26^ Dividing and separating peroxisomes by motor forces/pulling in these cell models is only possible when the organelles are tethered (eg, to other organelles or the cytoskeleton) as otherwise they would simply move to the cell periphery as observed in COS7 cells. These findings indicate that peroxisome tethering is cell type specific, and that the degree of tethering (in close interplay with the motor machinery/motile forces) modulates peroxisome distribution and proliferation. Interestingly, live cell imaging revealed that peroxisome motility is higher in COS7 cells than in human skin fibroblasts.^25^, ^34^ In line with this, ultrastructural studies indicate a lower degree of peroxisome‐ER association in COS7 cells.

We also investigated the effect of MIRO1‐mediated motor/pulling forces in cellular models of peroxisome disease.^25^, ^26^ Loss of the peroxisomal matrix protein import receptor PEX5 is associated with Zellweger Syndrome, a severe peroxisome biogenesis disorder with several developmental and neurological abnormalities.^79^ On the cellular level, PEX5‐deficient peroxisomes are import‐incompetent for matrix proteins, with a loss of their metabolic functions. Patient fibroblasts contain a reduced number of enlarged peroxisomal membrane structures (so called ghosts), which are “empty,” as peroxisomal matrix enzymes accumulate in the cytoplasm and/or are degraded. Remarkably, expression of MIRO1‐Pex in PEX5‐deficient fibroblasts resulted in the formation of highly elongated peroxisomal membrane protrusions, which associated with microtubules.^25^, ^26^ Again, protrusion formation and membrane elongation is only possible when the peroxisomes are tethered. Furthermore, calculations based on ultrastructural and live cell imaging data indicate that the surface area of the spherical peroxisomes is far too low to give rise to the extended membrane protrusions without phospholipid transfer from another source.^25^, ^26^ These observations further support the notion that peroxisome‐ER MCSs tether peroxisomes to the ER and mediate phospholipid transfer to allow peroxisomal membrane elongation and protrusion formation (see Section 2.1.2). Such a scenario would indicate that the phospholipid transfer between the ER and peroxisomes is much more dynamic than previously anticipated.

Detailed studies on the regulation of peroxisome positioning by MCSs have been performed in the yeast *S. cerevisiae*. In baker's yeast, peroxisomes move along actin filaments. The actin‐dependent myosin V motor Myo2 is recruited to peroxisomes by Inp2 (Inheritance protein 2), a peroxisomal single pass membrane protein. This is essential for the proper partitioning of peroxisomes between mother and daughter cells during asexual reproduction by budding, and thus for peroxisome inheritance. For balanced distribution, Inp1 (Inheritance protein 1), a peripheral membrane protein, links peroxisomes to the peripheral ER, thus ensuring that some peroxisomes are retained in the mother cell (reviewed in References 80, 81). Here, the MCS is composed of Pex3, a peroxisomal membrane protein, which distributes to peroxisomes and the ER, and is bridged by Inp1.^82^

### 2.3 Towards the identification of additional peroxisome‐ER tethers

#### ACBD4‐VAPs

It is very likely that besides ACBD5‐VAPs and Pex3‐Inp1, other proteins contribute to peroxisome‐ER tethering in mammalian and yeast cells. A machine learning prediction approach revealed the peroxisomal localization of ACBD4 (isoform 2).^29^ Like ACBD5, ACBD4 (isoform 2) is also a C‐tail‐anchored membrane protein with an N‐terminal acyl‐CoA‐binding domain. Although ACBD4 and ACBD5 share 58% sequence identity, this is mainly due to similarities in the N‐terminal acyl‐CoA‐binding domain, with the rest of the proteins showing significant differences. ACBD4 interacts with ER‐resident VAPs via a FFAT‐like motif (Figure 3), and co‐expression of ACBD4 and VAPB increased the number and surface of peroxisome‐ER MCSs, indicating that ACBD4 also participates in ER‐peroxisome MCSs in mammalian cells.^30^

#### MOSPD2‐ACBD5/4

Very recently, motile sperm domain‐containing protein 2 (MOSPD2) was identified as a novel ER tether in a proteomics approach.^36^ Like ER‐resident VAPs, MOSPD2 is an ER‐anchored protein which possesses an MSP domain (Figure 3). It interacts with several FFAT‐containing tether proteins from endosomes, mitochondria, or Golgi, thus mediating the formation of MCSs by bridging the ER with a variety of distinct organelles. As MOSPD2 can interact with a variety of tether proteins containing a FFAT‐motif (eg, mitochondrial PTPIP51, endosomal ORP1L and STARD3, or Golgi STARD11), it is possible that MOSPD2 also interacts with peroxisomal ACBD5 and ACBD4, which both possess FFAT‐like motifs (Figure 3). However, experimental evidence for a role of MOSPD2‐ACBD5/4 in ER‐peroxisome tethering is yet lacking.

#### FIS1‐BAP31

B‐cell receptor‐associated protein 31 (BCAP31/BAP31), an abundant 28‐kDa integral membrane chaperone protein of the ER and ER protein‐sorting factor, has been shown to function in ER‐mitochondria tethering by interacting with mitochondrial FIS1.^38^ FIS1‐BAP31 form a mitochondria‐ER platform which is crucial for the recruitment and activation of procaspase 8 and the conveyance of the apoptotic signal from mitochondria to ER.^38^ As the FIS1‐BAP31 interaction is also present in normal, nonapoptotic cells, it is suggested that the FIS1‐BAP31 hub may have other roles than apoptotic signaling. Interestingly, FIS1, a C‐tail anchored membrane protein, is also targeted to peroxisomes, where it contributes to peroxisomal division.^39^, ^83^ Several components of the division machinery at the outer mitochondrial membrane such as the C‐tail anchored membrane adaptor MFF and the fission GTPase DRP1 are also involved in peroxisomal division (reviewed in References 9, 20) (see Section 2.1.2). The adaptor proteins MFF and FIS1 recruit DRP1 to the organelle membrane, which oligomerizes into ring‐like structures, and upon GTP hydrolysis mediates membrane scission. As FIS1 is also a peroxisomal protein, it could as well contribute to peroxisome‐ER MCSs by interaction with BAP31 (Figure 3). Interestingly, the C‐tail anchored anti‐apoptotic proteins BCL‐XL and BCL‐2 can target peroxisomes.^29^ The physiological role of these proteins on peroxisomes awaits clarification. However, a recent study revealed that loss of VDAC2 shifts the localization of BAK, a pro‐apoptotic member of the BCL‐2 family, from mitochondria to peroxisomes and the cytosol, thereby leading to a release of peroxisomal matrix proteins including catalase to the cytosol.^84^ A subset of BAK seems to localize to peroxisomes in control cells, regulating peroxisomal membrane permeability and catalase localization.^84^ Cytosolic catalase can protect against H_2_O_2_‐mediated redox changes and may constitute a cellular defense mechanism to combat oxidative insults of extra‐peroxisomal origin.^85^

### 2.4 ACBD5 deficiency—a novel peroxisomal disorder with an accumulation of VLCFAs

Besides its FFAT‐like motif, which mediates peroxisome‐ER MCSs, ACBD5 also possesses an N‐terminal acyl‐CoA‐binding domain implying a function as an intracellular carrier of acyl‐CoA esters (Figure 3). The first patients diagnosed with a genetic ACBD5 deficiency were three siblings presenting with retinal dystrophy and white matter changes.^86^ Further characterization of those and another patient revealed a peroxisome‐based disorder with progressive leukodystrophy, ataxia, progressive microcephaly with facial dysmorphisms, in addition to retinal dystrophy.^14^, ^87^ In all cases, ACBD5 protein was absent due to a homozygous splice site mutation^86^ or a deleterious homozygous mutation deleting exons 7 and 8, causing a premature stop codon.^14^ The main biochemical feature of ACBD5 deficiency is the accumulation of very long chain fatty acids (VLCFAs). Analysis of peroxisomal parameters in blood revealed an abnormal VLCFA profile and accumulation of C26:0 lysoPC.^14^ Studies with patient's skin fibroblasts confirmed an abnormal VLCFA profile with increased concentration of C26:0. Peroxisomal C26:0 β‐oxidation activity was reduced, but pristanic acid oxidation activity was normal (excluding a general defect in peroxisomal β‐oxidation). Furthermore, accumulation of D3‐C26:0 and D3‐C28:0 after loading of fibroblasts with D3‐C22:0 was detected, indicating increased chain elongation due to increased substrate availability.^14^ Peroxisome integrity and the import of membrane or matrix proteins were not affected. The phenotype of ACBD5‐deficient fibroblasts was also recapitulated in ACBD5‐KO HeLa cells generated via the CRISPR/Cas9 system.^14^, ^87^ Overall, these findings clearly show that ACBD5 deficiency is a novel single peroxisomal protein/enzyme deficiency causing an impaired VLCFA metabolism.

**Figure 4:**
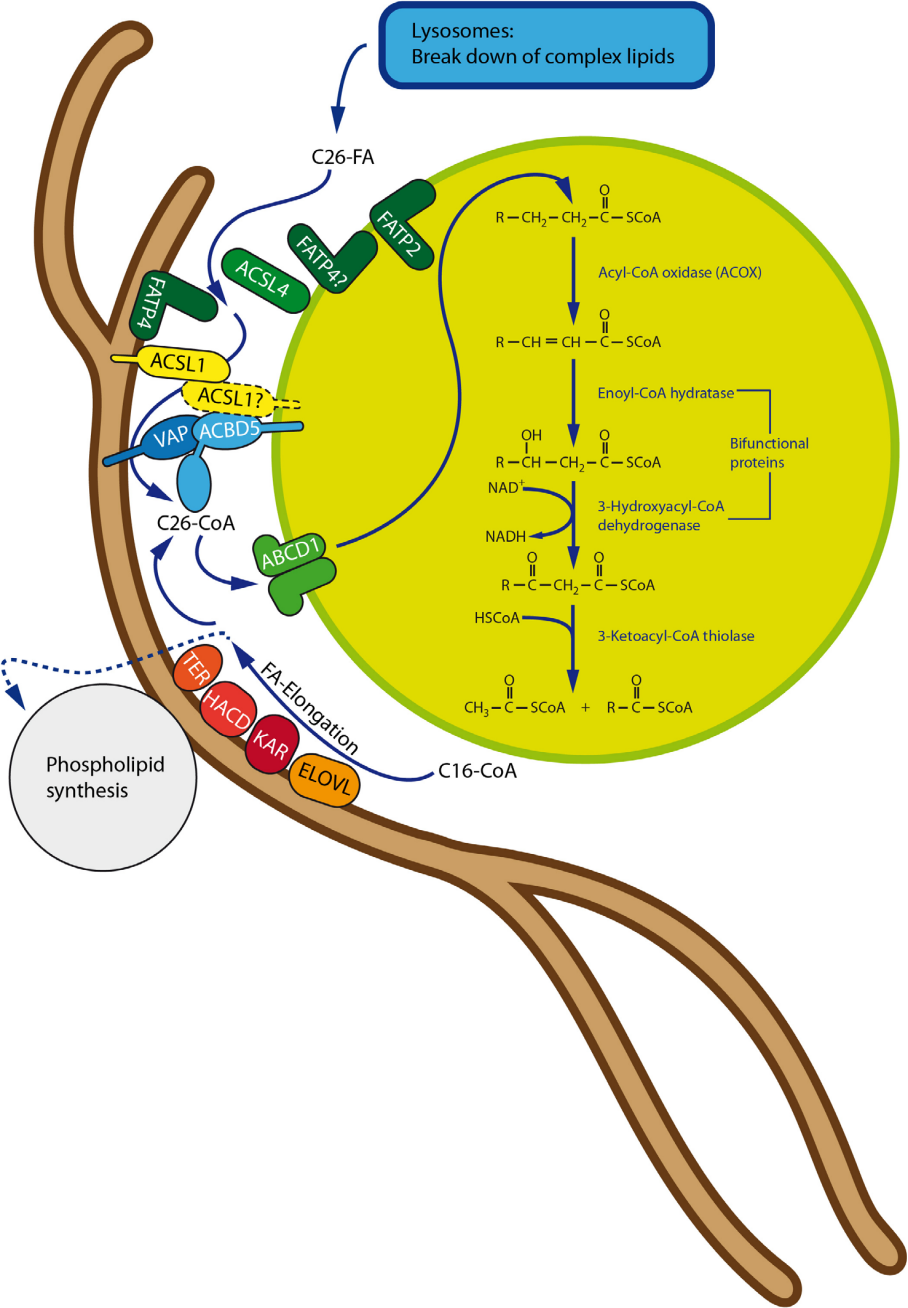
Interplay between peroxisomal VLCFA degradation and ER fatty acid elongation at ACBD5‐mediated MCSs at the PO‐ER interface. At the ER membrane, palmitoyl‐CoA (C16) is elongated to form saturated VLCFA‐CoA in four subsequent steps by the enzymes ELOVL(1‐7) (elongation of very‐long chain fatty acids protein), KAR (3‐ketoacyl‐CoA reductase), HACD(1‐3) (very‐long‐chain‐3‐hydroxyacyl‐CoA dehydratase), and TER (trans‐2,3‐enoyl‐CoA reductase). VLCFAs are subsequently incorporated into membrane lipids at the ER or, when VLCFA concentrations in the membrane are already high, transferred to peroxisomal ACBD5. Long and very‐long chain fatty acid‐CoA synthetases (ACSL and FATP) generate fatty acyl‐CoA from the cytosolic free fatty acid pool. ACBD5 “senses” growing concentrations of VLCFA‐CoA at the contact site by binding to its ACB domain (probably by direct interaction with, for example, ACSL1). Bound VLCFA‐CoA is “handed over” to the peroxisomal FA import protein ABCD1 to be imported and degraded by the peroxisomal β‐oxidation pathway. Such a regulatory system may prevent excessive incorporation of VLCFA into phospholipids, which are as well generated at the ER

VLCFAs enter the peroxisomes via ABCD1, a member of the **A**TP **B**inding **C**assette Subfamily **D** family^88^ (Figure 4). Defects in ABCD1 are the cause of X‐linked adrenoleukodystrophy (X‐ALD).^89^ It has been suggested that ACBD5 facilitates transport of VLCFA‐CoAs into peroxisomes for subsequent β‐oxidation.^14^ ACBD5 is likely involved in capturing C26‐CoA in the cytosol through its acyl‐CoA‐binding domain and presenting it to the VLCFA transporter ABCD1. ABCD1 then transports the C26‐CoA into the peroxisome where it is β‐oxidized by the sequential action of the peroxisomal β‐oxidation enzymes acyl‐CoA oxidase 1 (ACOX1), D‐bifunctional protein (DBP), sterol‐carrier protein X, and 3‐ketoacyl‐CoA thiolase^14^, ^87^ (Figure 4). In line with this, a preference for ACBD5 to bind VLCFA‐CoAs has been reported.^87^

**Figure 5:**
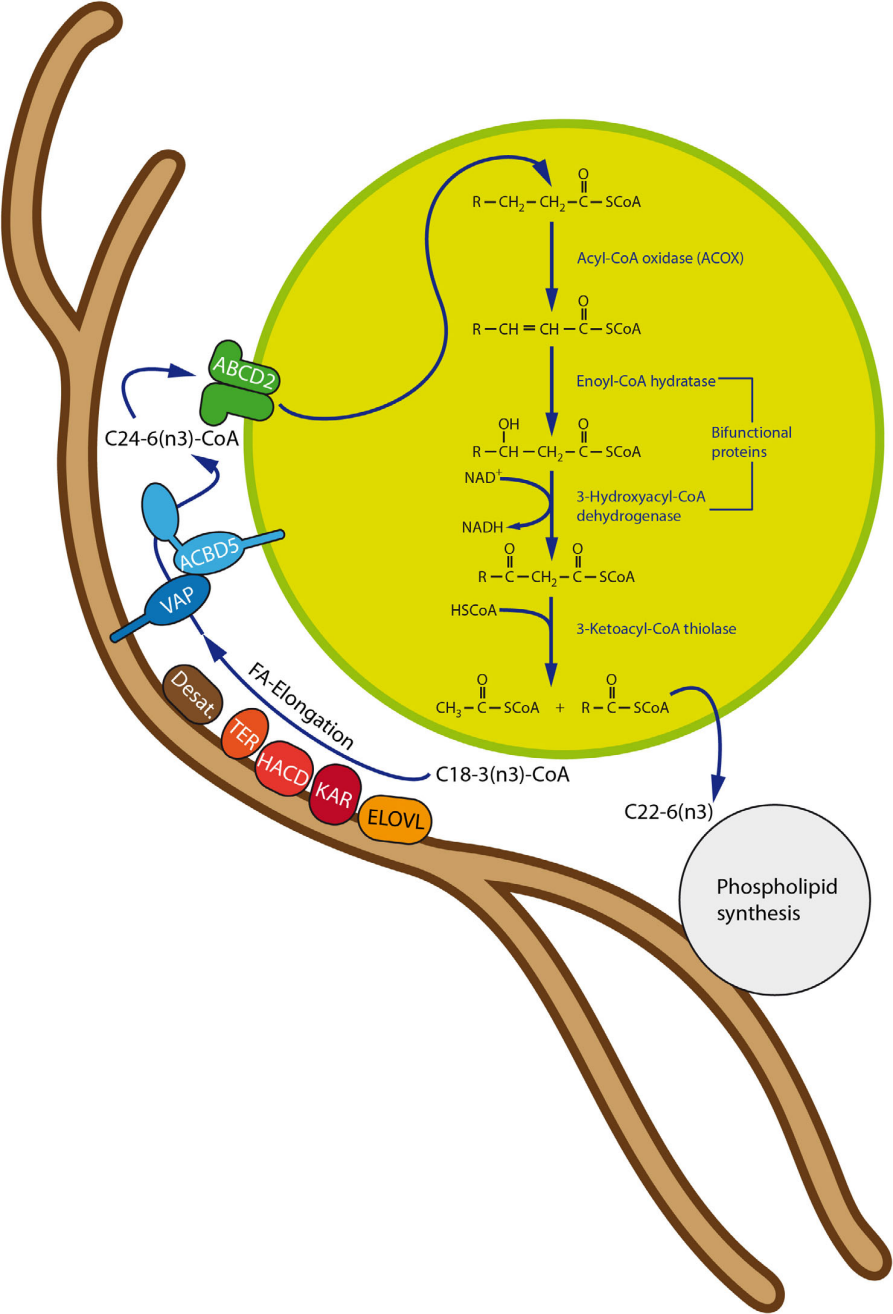
Peroxisome‐ER cooperation in PUFA synthesis at ACBD5‐mediated MCSs. PUFAs are synthesized at the ER by combined FA elongation and desaturation. However, peroxisomes cooperate with the ER in the synthesis of n‐3 long‐chain PUFAs such as docosahexaenoic acid (DHA) (22:6n‐3) as the ER appears to lack potent fatty acid Δ4‐desaturase activity. To this end, exploiting Δ6 desaturase activities, the ER produces 24:6 (n‐3) FA, which are subsequently chain‐shortened by one round of β‐oxidation in peroxisomes. In this scenario, ACBD5 may facilitate efficient transport of 24:6 (n‐3) CoA from the ER to peroxisomes at MCSs. PUFA, polyunsaturated fatty acid

VLCFA accumulation as the dominant biochemical abnormality is also observed in the peroxisomal disorders X‐ALD and ACOX1‐deficiency.^79^ ACBD5‐deficient patients develop clinical symptoms such as progressive leukodystrophy, ataxia, retinal dystrophy, cleft palate, and facial dysmorphism, which resemble those of patients suffering from ACOX1 deficiency, but are different from X‐ALD, where, for example, retinopathy does not manifest. In addition, magnetic resonance imaging (MRI) abnormalities in ACBD5‐deficient patients are distinct from those seen in X‐ALD.^90^, ^91^ An underlying reason for these differences (besides potential differences in expression pattern, partial complementation by related proteins, or accumulation of different phospholipid species containing VLCFAs) may be the additional role of ACBD5 in the formation of peroxisome‐ER MCSs. It is possible that ACBD5‐mediated peroxisome‐ER MCSs contribute to the formation of a peroxisome‐ER hub to coordinate fatty acid metabolism at both organelles (Figures 4, 5). The ER is the main site of VLCFA synthesis such as C26:0‐CoA from shorter‐chain fatty acids (synthesized from acetyl‐CoA followed by their conversion into C16:0‐CoA by the Fatty Acid Synthase [FAS]‐complex in the cytosol), followed by chain elongation at the ER membrane by a multienzyme complex including ELOVLs (elongation of very‐long chain fatty acids protein)^92^ (Figure 4). Proximity of the organelles and membrane‐bound proteins involved in fatty acid metabolism within the peroxisome‐ER hub may allow coordinated channeling of fatty acids either towards elongation (and use in esterification reactions or PUFA synthesis; see Section 2.5) or degradation by peroxisomal β‐oxidation (eg, when VLCFA concentration is too high) (Figure 4). In a recent BioID study, the long‐chain acyl‐CoA synthetase ACSL1 was identified as a direct interaction partner of ACBD5 and VAPB.^93^ In the liver, about 50% of ACSL1 is located on the ER indicating that ACSL1 is present at the ER‐peroxisome interface to activate fatty acids destined for peroxisomal metabolism (Figure 4). ACSL1 can activate branched‐chain fatty acids such as phytanic acid, which is derived from dietary phytol.^94^, ^95^ Phytanic acid is degraded in peroxisomes by α‐ and β‐oxidation. In line with this, ACSL1 was found to interact with proteins of the phytol metabolic pathway such as fatty aldehyde dehydrogenase (FALDH) and ABCD3, the peroxisomal transporter for branched‐chain fatty acids. Although the functional role of the interaction of ACSL1 with VAPB and ACBD5 remains unclear, the findings are supportive of a peroxisome‐ER hub which may be crucial for the capture, activation and channeling of fatty acids to coordinate lipid metabolism at the ER‐peroxisome interface (Figure 4).

### 2.5 Peroxisome‐ER MCSs and their role in cellular ether‐phospholipid biosynthesis

It is well known that peroxisomes and the ER cooperate metabolically in the biosynthesis of ether‐phospholipids, which is initiated in peroxisomes and completed in the ER.^96^ Ether glycerolipids constitute about 15% to 20% of total cellular membranes and are particularly enriched in brain (eg, myelin sheath lipids), heart and immune cells of the blood. Plasmalogens, a class of ether phospholipids, represent ∼20% of the total phospholipid mass in humans.^96^ Ether phospholipids are supposed to contribute to a reduction in membrane fluidity^97^, ^98^ and to act as ROS scavengers preventing the oxidation of other membrane lipids.^99^ Patients with a defect in ether‐phospholipid biosynthesis (eg, due to DHAPAT [GNPAT] or ADHAPS [AGPS] deficiency; see below), present with Rhizomelic Chondrodysplasia Punctata (RCDP) characterized by skeletal dysplasia, severe central nervous system (CNS) abnormalities, cortical cataracts, and poor survival with most patients dying before reaching adulthood.^96^ On the cellular level, ether phospholipid deficiency affects membrane traffic and cholesterol distribution as well as the integrity of the plasma membrane.^100^, ^101^ Furthermore, the peroxisomal steps in ether phospholipid biosynthesis are important for the formation of glycosylphosphatidylinositol (GPI)‐anchored proteins in the ER.^102^

In higher eukaryotes, alkyldihydroxyacetone phosphate synthase (ADHAPS) is the only enzyme able to catalyze the formation of the characteristic ether‐bond in ether‐phospholipids. ADHAPS forms a heterotrimeric enzyme complex with dihydroxyacetone phosphate acyltransferase (DHAPAT) at the inner site of the peroxisomal membrane.^103^ DHAPAT catalyzes the acylation of dihydroxyacetone phosphate (acyl‐DHAP) from glycerone‐3‐phosphate and an acyl‐CoA ester. ADHAPS then substitutes the acyl group with an alkyl group. Additional reactions providing the long‐chain alcohol for the substitution reaction are performed by one of the two tail‐anchored acyl‐CoA reductases (FAR1 and FAR2), which are associated with the cytosolic site of the peroxisomal membrane. The three enzymes are indispensable for ether‐phospholipid biosynthesis.^104^, ^105^, ^106^ As all further reactions (acylation in position 2 of the glycerol, dephosphorylation, and addition of ethanolamine/cholin in position 3) are carried out in the ER, the alkyl‐DHAP needs to be shuttled from peroxisomes to the ER. Interestingly, loss of the ACBD5‐VAP tether by depleting either ACBD5 or VAPs has been reported to modestly reduce cellular PE‐plasmalogen levels in cultured HeLa cells.^35^ In line with this, both PE‐ and PC‐ether phospholipids were markedly decreased in skin fibroblasts from patients with ACBD5 deficiency, suggesting a general defect in ether phospholipid biosynthesis, including plasmalogens.^107^ These findings led to the suggestion that peroxisome‐ER MCSs, in particular those mediated by the ACBD5‐VAP tether, play a role in alkyl‐DHAP shuttling and efficient ether‐phospholipid biosynthesis. If ether‐phospholipid biosynthesis is also reduced in tissues of ACBD5‐deficient patients, and if a reduction in ether‐phospholipids also contributes to the neurological abnormalities, awaits further clarification.

Metabolic cooperation between ER and peroxisomes is also required for the generation of polyunsaturated fatty acids (PUFAs). This usually involves a series of chain‐elongation and desaturation reactions at the ER; however, as mammalian cells are supposed to lack an acyl‐CoA‐dependent delta‐4‐desaturase,^108^ direct synthesis is not possible, and C24:6 (n‐3) and C24:5 (n‐6) fatty acids synthesized in the ER need to be transferred to peroxisomes (Figure 5). It was recently suggested that human fatty acid desaturase 2 (FADS2) can perform delta‐4 desaturation,^109^ however, studies are mainly based on overexpression, and the physiological relevance in vivo remains unclear. After transfer to the peroxisomes, those fatty acids then undergo a single round of peroxisomal β‐oxidation to produce C22:6n‐3 and C22:5n‐6 and are transferred back to the ER for incorporation in different lipid species^110^, ^111^, ^112^ (Figure 5). This metabolic ER‐peroxisome interplay is particularly important for the synthesis of docosahexaenoic acid (DHA, C22:6n‐3), a major n‐3 PUFA in adult mammalian brain and retina, which can be produced endogenously from linolenic acid (C18:3 n‐3). DHA deficiency results in memory loss, learning disabilities, and impaired vision.^113^ In patients with ACOX1‐ and DBP‐deficiency and subsequent defects in peroxisomal β‐oxidation, DHA is known to be decreased.^111^ Interestingly, DHA/DHA‐containing phospholipids are also required for the plasticity and membrane dynamics of peroxisomes, for example, for membrane elongation and division.^114^ In a recent lipidomics study, a variety of phospholipid species containing PUFAs (including DHA) were found to be decreased in ACOX1‐ and DBP‐deficient fibroblasts, but not in fibroblasts from ALD patients. The peroxisome‐dependent synthesis of DHA in ACBD5‐deficient fibroblasts appeared to be normal.^87^

## 3 PEROXISOME‐MITOCHONDRIA CONTACTS AND FUNCTIONAL INTERPLAY

It is well established that peroxisomes and mitochondria are functionally connected (for recent reviews see References 115, 116, 117, 118). This interplay, termed the “peroxisome‐mitochondria connection”^9^, ^19^, ^117^, ^119^ comprises the metabolic cooperation of peroxisomes and mitochondria (eg, in the β‐oxidation of fatty acids, phytanic acid α‐oxidation, bile acid synthesis, and glyoxylate detoxification) (reviewed in Reference 18) (Figure 2), peroxisome‐mitochondria cooperation in cellular redox balance and redox signaling (reviewed in References 120, 121), cooperation in anti‐viral signaling and combat,^122^, ^123^ as well as coordinated biogenesis by sharing of key proteins of their division machinery.^20^, ^117^, ^124^ Several key division proteins are C‐tail anchored membrane proteins which are dually targeted to peroxisomes and mitochondria.^29^, ^44^, ^124^, ^125^ Mitochondria can also contribute to the biogenesis (de novo formation) of peroxisomes under certain experimental conditions.^126^, ^127^ In addition, dysfunctional mitochondria were reported in several peroxisomal disorders^117^, ^128^, ^129^ highlighting the physiological importance of peroxisome‐mitochondria interplay, in particular in hepatocytes.

Peroxisomes catalyze the β‐oxidation of a variety of substrates including saturated VLCFA (eg, hexacosanoic acid [C26:0]), branched‐chain fatty acids (eg, pristanic acid), the bile acid intermediates di‐ and tri‐hydroxycholestanoic acid (DHCA and THCA), and long‐chain dicarboxylic acids.^130^, ^131^ Whereas fatty acid β‐oxidation in yeast and plants is solely peroxisomal, in mammals both peroxisomes and mitochondria possess their separate β‐oxidation pathways. Some fatty acid substrates (eg, VLCFA) are unique to peroxisomes as they cannot be degraded by mitochondria. However, peroxisomal β‐oxidation only results in chain‐shortened fatty acids (medium chain acyl‐CoA, C6‐C8), which have to be shuttled to mitochondria for full oxidation to CO_2_ and H_2_O. Furthermore, acetyl‐CoA and nicotinamide adenine dinucleotide (NADH) generated by peroxisomal β‐oxidation need to be routed to mitochondria, in particular for energy‐efficient reoxidation of NADH back to NAD+.^118^ The α‐oxidation of 3‐methyl branched‐chain FAs is another unique function of peroxisomes. Phytanic acid, a dominant 3‐methyl branched‐chain FA in our diet, for example, can only undergo β‐oxidation after the terminal carboxyl group is released by α‐oxidation. Alpha‐oxidation in peroxisomes relies on a close interplay with mitochondria, for example, for the provision of 2‐oxoglutarate (required in the phytanoyl‐CoA hydroxylase reaction), the reoxidation of NADH (generated by pristanal dehydrogenase) and the provision of ATP (required by pristanoyl‐CoA synthetase) (reviewed in References 18, 132).

Molecular tethers which link peroxisomes to mitochondria have recently been identified in baker's yeast by a systematic screening approach using a proximity detection method based on split fluorophores.^3^ Individual tethering functions for the yeast mitofusin Fzo1 and the peroxisomal membrane protein Pex34 in peroxisome‐mitochondria MCSs were revealed. Pex34 interacts with Pex11 family peroxins and is involved in the control of peroxisome morphology and abundance.^133^ Importantly, the study also demonstrated a physiological role for peroxisome‐mitochondria MCSs in linking peroxisomal β‐oxidation and mitochondrial ATP generation by the citric acid cycle.^3^ Peroxisome proximity to mitochondria was observed to increase based on fluorescence microscopy when yeast cells were grown on oleate as the sole carbon source. The involvement of Pex34‐mediated peroxisome‐mitochondria MCSs in the transfer of β‐oxidation products was demonstrated biochemically by incubating the yeast cells with radiolabeled [1‐C14] octanoate (C8:0) and measuring the rate of acetyl‐CoA transfer to mitochondria and its conversion to CO_2_. Overexpression of Pex34 (but not of Fzo1) resulted in a marked increase in CO_2_ production indicating that Pex34‐mediated expansion of MCSs stimulated the transport of acetyl‐CoA from peroxisomes to mitochondria.^3^ The increased CO_2_ production in the Pex34 overexpressing cells was abolished by deleting citrate synthase and only reduced when deleting acetylcarnitine transferase. This indicates that peroxisomal acetyl‐CoA is predominantly exported to mitochondria via conversion into citrate by peroxisomal citrate synthase, and not via the carnitine pathway.^3^ Pex34 and Fzo1 likely act in different tether complexes at peroxisome‐mitochondria MCSs, which have different functions.

In baker's yeast, peroxisomes have also been reported to localize adjacent to a specific mitochondrial niche near the ER‐mitochondria MCS, proximal to where the pyruvate dehydrogenase complex is located in the mitochondrial matrix.^134^ This suggests a three‐way organelle junction. Peroxisomal Pex11 and mitochondrial Mdm34, a protein of the ER‐mitochondria tether (ERMES), are reported to mediate the peroxisome‐mitochondria MCS.^134^

In contrast to yeast, little information is available on MCS between peroxisomes and mitochondria in mammalian cells (Figure 2). In addition to the pathways mentioned above, mammalian peroxisome‐mitochondria interplay appears to be required for hormone‐induced, controlled steroid hormone biosynthesis. Immunofluorescence and live‐cell studies with MA‐10 mouse tumor Leydig cells revealed that treatment with di‐butyryl‐cAMP induced peroxisomes to approach mitochondria.^135^ It is suggested that the acyl‐CoA‐binding protein ACBD2/ECI2 inserts head to tail into peroxisomes and mitochondria. This interaction between both organelles may contribute to the supply of cholesterol used for steroid hormone biosynthesis.^135^

## 4 PEROXISOME‐LYSOSOME CONTACTS AND CHOLESTEROL TRAFFICKING

Out of the interactions between peroxisomes and other organelles, lysosomal contacts were the last to be recognized. Indeed, peroxisomal membrane contacts with lysosomes (LPMC) appear to be less frequent than those with the ER or mitochondria, but still comprise around 15% to 20% of the total peroxisome number in mammalian cells.^4^, ^49^ However, as LPMC formation is of transient nature, contact frequencies can change in response to different stimuli. Incubation of Hela cells with LDL (low‐density lipoprotein), delivering lipids to the cell, increased LPMC in a time‐dependent manner implying that LPMC form in a tightly controlled process in order to transfer specific metabolites.^49^ The metabolic significance of LPMCs was detected in an RNAi screen for genes associated with lysosomal cholesterol transport deficiencies.^49^ Cholesterol is synthesized de novo by a pathway shared by the ER, mitochondria and the cytosol, or taken up by food consumption where it is delivered to the target cells via LDL, which under normal conditions is the dominant route of cellular cholesterol supply. After LDL uptake by plasma membrane LDL receptors, LDL‐contained lipids are transported via endosomes to lysosomes, where cholesterol is liberated by acid lipase from the cholesteryl esters and targeted to the ER for redistribution into the different subcellular membranes.^136^, ^137^ Unexpectedly, knockdown of a considerable number of peroxisomal genes led to the accumulation of cholesterol in lysosomes indicating that peroxisomes might be involved in this cellular cholesterol transport system.^49^ Subsequently, the authors showed that synaptotagmin VII (Syt7) on lysosomes forms specific interactions with phosphatidylinositol 4,5‐bisphosphate (PI(4,5)P_2_) in the peroxisome membrane and is required for a functional cholesterol transport from lysosomes to peroxisomes (Figure 2). It should be noted that some of the technical aspects of the paper are critically debated, for instance, the use of anti‐SKL‐antibodies to pull down peroxisomes (as proteins with a C‐terminal serine‐lysine‐leucine (SKL) targeting signal are imported into the peroxisome lumen). According to these findings, Song et al proposed a novel route for the intracellular cholesterol transport: after import of LDL‐bound cholesterol esters and enzymatic hydrolysis in lysosomes, cholesterol is bound by lysosomal Niemann‐Pick C2 and C1 protein (NPC2, NPC1) and subsequently delivered to peroxisomes via the LPMCs. At peroxisomes, cholesterol could be incorporated into the peroxisome membrane or redistributed to other organelles like the ER using further organelle contact sites. As peroxisomes also perform the last steps in bile acid formation—the major route of cholesterol depletion—they might in this way act as a central sensory hub regulating intracellular cholesterol distribution. Indeed, decreased levels in cellular cholesterol after knockdown of the peroxisome‐ER tethering protein ACBD5 may point to such a central position in cholesterol trafficking.^35^ Mutations in VAPB have been linked to amyotrophic lateral sclerosis (ALS).^15^ As ALS patients carrying a VAPB (P56S) mutation are reported to have increased cholesterol levels,^138^ it was speculated that this increase may be caused by increased ER‐peroxisome contacts.^35^ Further studies on a possible role of the ER‐peroxisome tethering in the pathogenesis of ALS are required.

Proteins which actively deliver cholesterol from lysosomes to peroxisomes have not yet been identified. Specific StAR‐related lipid transfer (START) domain proteins (STARDs), OSBPs, and OSRPs may be involved in this nonvesicular trafficking pathway.^136^ While such molecular details have still to be revealed, a recent follow‐up study by the Song‐group reports novel insights into the regulatory mechanism which could facilitate dynamic interactions of peroxisomes and lysosomes at the LPMCs.^139^ In this work, the authors searched for enzymatic regulators of phosphatidylinositol phosphate (PIP) kinases producing PI(4,5)P_2_ from PI(4,5)P using an RNAi approach. Knockdown of a single enzyme, PIP4K2A, was found to induce lysosomal cholesterol accumulation and reduced LPMC formation via decreasing PI(4,5)P_2_ levels.^139^ Using an in vitro LPMC reconstitution assay, the authors reported that PIP4K2A has to specifically reside at peroxisomes to produce PI(4,5)P_2_. It remains to be clarified if the protein is imported into peroxisomes or if it localizes to specific binding partners at the outer surface of the peroxisomal membrane.

With regard to this potential role in intracellular cholesterol transport, it is consequent to ask if the cellular cholesterol homeostasis is disturbed in peroxisomal disorders. Song et al measured cholesterol concentrations in fibroblasts of Zellweger spectrum disorder (ZSD) and X‐linked adrenoleukodystrophy patients and found a remarkable accumulation of cholesterol in all cases.^49^ While this could be straight‐forwardly explained by a general malfunction of peroxisome metabolism in ZSD, the accumulation of cholesterol in ABCD1 deficiency remains unexplained. However, the observed reduction in LPMCs after ABCD1 knockdown is consistent with the increase in cholesterol in patient cells, tissues from knockout mice and silenced cells.

Kassmann et al observed an accumulation of lysosomes juxtaposed to peroxisomes at the paranodal loops of axons in Schwann‐cell−/oligodendrocyte‐specific and general peroxisomal KO mouse models including the Abcd1−/mouse.^140^ These lysosomes colocalized with ganglioside aggregates enriched in VLCFA suggesting a disruption of the interplay between peroxisomal and lysosomal lipid catabolism. Thus, the interaction between peroxisomes and lysosomes appears to include the exchange of several lipid metabolites between both organelles which may result in a general dysregulation in LPMC formation. Moreover, accumulation of nonfunctional lysosomes in parallel to a decrease in peroxisomes was recently reported after suppression of the mitochondrial and peroxisomal fission factor FIS1 in chondrocytes.^141^ The knockdown of FIS1, resulting in a disturbed lipid metabolism, was further accompanied by dysregulation in micro‐RNAs targeting lysosomal function, indicating that the interplay between peroxisomes and lysosomes may be regulated at various different levels. Therefore, the unexpected accumulation in cholesterol in ABCD1‐deficient cells may be a secondary response to a nonfunctional interplay between peroxisomes and lysosomes, which represent in a lysosomal storage disease‐like phenotype. In this regard, in addition to mitochondria and ER‐contacts, the LPMCs appear to establish as another complex and tightly regulated interaction network, which requires further research to decipher individual molecular components and regulatory pathways to understand its role in the complicated pathology of peroxisomal disorders.

## 5 PEROXISOME‐LIPID DROPLET CONTACTS AND FUNCTIONAL INTERPLAY

Lipid droplets are dynamic organelles which contribute to the storage of neutral lipids such as triacylglycerols and sterol esters. Close associations of lipid droplets with other organelles including peroxisomes have been described (for recent reviews see References 10, 142, 143, 144, 145). A system‐level analysis of the organelle interactome in COS‐7 cells using a multispectral image acquisition method revealed that 10% of lipid droplets made contact with peroxisomes.^4^ Interestingly, the fraction of lipid droplet‐peroxisome contacts decreased (and that of lipid droplet‐lysosome contacts increased) after treatment with excess oleic acid.^4^ This is likely due to increased lysosomal digestion of lipid droplets under excess oleic acid conditions.^146^ The peroxisome‐lipid droplet interaction may link lipolysis mediated by lipid droplets to peroxisomal fatty acid β‐oxidation; furthermore, lipids generated by peroxisomes may move into lipid droplets (reviewed in Reference 19) (Figure 2). In addition, deficiency of peroxisomal β‐oxidation or loss of peroxisomes has been associated with enlarged lipid droplets and alterations in their number.^147^, ^148^ In baker's yeast, peroxisomes form very intimate contact sites, termed pexopodia, with lipid droplets.^149^ Pexopodia protrude into the lipid droplet core and are enriched in components of the β‐oxidation machinery indicating that they might stimulate neutral lipid breakdown and transfer of fatty acids from the lipid droplet to the peroxisome. Although there are various reports about the interaction between peroxisomes and lipid droplets, peroxisome‐lipid droplet tether proteins are still scarce. An interactome map of protein‐protein interactions between peroxisomes and lipid droplets in baker's yeast revealed that ERG6 and PET10, which reside in lipid droplets, interact with several peroxisomal proteins.^150^ Whether these proteins constitute a genuine tether requires further investigation. Interestingly, in baker's yeast, newly formed lipid droplets and peroxisomes remain associated with conserved ER subdomains, suggesting a link between lipid droplet and peroxisome biogenesis.^151^ Very recently, a role for the hereditary spastic paraplegia protein M1 Spastin, a membrane‐bound AAA ATPase on lipid droplets, in the tethering of lipid droplets to peroxisomes was revealed.^13^ Interestingly, M1 Spastin forms a tethering complex with the peroxisomal fatty acid transporter ABCD1 to promote lipid droplet‐peroxisome MCSs. The interaction depends on a peroxisome‐interacting region (amino acids 197‐328) in M1 Spastin, and its N‐terminal hairpin motif, which inserts directly into the lipid monolayer of the lipid droplet. Furthermore, the ATPase activity of M1 Spastin can regulate lipid droplet‐peroxisome MCSs, suggesting a link to fluctuations in cellular ATP levels. The authors also show that the M1 Spastin‐ABCD1 complex works in conjunction with the ESCRT‐III (endosomal sorting complexes required for transport) proteins IST1 (increased sodium tolerance 1) and CHMP1B (charged multivesicular body protein 1B) in the delivery of fatty acids from lipid droplets to peroxisomes.^13^ The membrane‐shaping proteins IST1 and CHMP1B are recruited via the M1 Spastin MIT (microtubule interacting and trafficking) domain and may facilitate fatty acid trafficking through modification of lipid droplet membrane morphology. The Spastin‐mediated trafficking of fatty acids to peroxisomes also prevents the accumulation of peroxidated lipids in lipid droplets. These exciting findings may help to shed light on the pathogenesis of diseases associated with defective fatty metabolism in lipid droplets and peroxisomes.

## 6 PEROXISOME‐PEROXISOME AND PEROXISOME‐PLASMA MEMBRANE CONTACTS AND FUNCTIONAL INTERPLAY

Live cell studies revealed that peroxisomes, which often move along microtubules in the cell, also self‐interact in transient and long‐term contacts.^19^, ^68^ The physiological role for this interaction is still unknown, but peroxisome‐peroxisome contacts may promote efficient metabolite exchange (eg, H_2_O_2_ or other ROS) and prevent leakage^11^, ^19^ (Figure 2). In this respect, it may be speculated that such peroxisome‐peroxisome interactions are part of a cellular “signaling system” to monitor the distribution and state of peroxisomes ensuring maintenance of the peroxisome population.^19^ The molecular mechanisms mediating peroxisome self‐interaction have yet to be revealed.

Budding yeast cells always retain some fraction of an organelle population in the mother cell and organelle retention requires MCSs/tethers among the organelles.^81^ In baker's yeast, Inp1 and Pex3 tether peroxisomes to the cortical ER for coordinated inheritance (see Section 2.2). These results suggested that peroxisomes interact indirectly with the plasma membrane via the cortical ER. Evidence for a direct interaction between peroxisomes and the plasma membrane in baker's yeast was recently provided using a proximity detection method based on split fluorophores.^3^ Whether this interaction also depends on Inp1 and Pex3, or involves other tether proteins, requires further clarification. Interactions between peroxisomes and the plasma membrane in human or animal cells have not yet been described.

### 6.1 Perspectives

Peroxisomes cannot function as isolated entities. As their metabolic functions require cooperation and exchange of metabolites with other organelles, they are integrated into a complex network of interacting organelles, which is now beginning to emerge. The molecular mechanisms and tethering components mediating peroxisome‐ER contacts have now been identified in mammalian cells, and important roles in peroxisome membrane expansion/biogenesis, mobility, positioning, and lipid metabolism have been revealed. The current efforts in the development of new screening approaches and techniques to characterize organelle contacts and associated proteins will certainly result in a more complete picture of peroxisome‐organelle contact sites and the molecules involved in tethering, which will further broaden our understanding of peroxisome cooperation and crosstalk with other compartments. A challenge ahead is to reveal their physiological functions and the mechanisms which mediate the transfer of phospholipids, metabolites, and signaling molecules between interacting organelles. Importantly, tethers often have additional functions in contact sites such as metabolite transfer. Addressing those challenges will require novel probes and tools, for example, to monitor and quantify phospholipid transfer, as well as cross‐discipline approaches combining molecular cell biology with biophysics, proteomics, lipidomics, metabolomics, and sophisticated imaging/quantification techniques. Important questions are how the formation of organelle contacts is regulated to control their dynamics, and if and how the number of organelle contacts is changing under different (patho)physiological conditions. The disruption of organelle contacts has been linked to disease, for example, to neurodegeneration,^152^ and the role and importance of MCSs in human health and disease is only starting to be revealed. A challenge is to diagnose MCS‐related diseases, where symptoms are likely complex and not well characterized, as tether proteins can have multiple functions, and tethering can be redundant with multiple protein complexes involved. With respect to peroxisomes and their central role in cellular metabolism, loss of contacts is suggested to impact on their optimal function. It will be challenging to diagnose functional changes in patients, as the metabolic functions of peroxisomes may only be slightly affected, and peroxisome morphology (including shape, number, size) may be normal. Thus, current diagnostic approaches which determine biomarkers (eg, VLCFA) in plasma or peroxisome morphology/protein localization in skin fibroblasts may miss patients with peroxisome MCS disorders. It is also suggested that organelle contacts, and their dynamic interplay, influences the development of common, age‐related disorders, for example, neurodegenerative diseases.^152^ A better understanding of organelle contacts may lead to therapeutic approaches allowing specific targeting and modulation of tethering complexes to combat degenerative diseases. This will require close cooperation between clinical, diagnostic, and fundamental research‐driven laboratories. Undoubtedly, research on organelle interplay and MCSs is an exciting, rapidly developing field, which will greatly impact on our understanding of human cell biology, health, and disease.

## CONFLICT OF INTEREST

The authors declare that they have no conflict of interest.

## AUTHOR CONTRIBUTIONS

M.S. and M.I. planned the manuscript. M.S., M.K., and M.I. wrote the manuscript and prepared the figures.
